# Spring-In Prediction of CFRP Part Using Coupled Analysis of Forming and Cooling Processes in Stamping

**DOI:** 10.3390/ma17051115

**Published:** 2024-02-28

**Authors:** Jae-Chang Ryu, Chan-Joo Lee, Jin-Seok Jang, Dae-Cheol Ko

**Affiliations:** 1 Department of Nanomechatronics Engineering, Pusan National University, Busan 46241, Republic of Korea; 2 Precision Manufacturing & Control R&D Group, Korea Institute of Industrial Technology, Jinju 52845, Republic of Korea; 3 Smart Manufacturing Technology R&D Group, Korea Institute of Industrial Technology, Daegu 42994, Republic of Korea

**Keywords:** carbon fiber reinforced plastic (CFRP), spring-in, stamping process, cooling process

## Abstract

The spring-in phenomenon of the composite parts can affect the assembly process. This study aims to predict the spring-in phenomenon of a carbon fiber reinforced plastic (CFRP) part. Here, we predict the spring-in of the CFRP part using a coupled analysis of the forming and cooling processes during the stamping process. First, a simulation of the entire forming process, such as the transfer of the composite laminate, gravity analysis, and forming was performed to obtain the temperature distribution of the CFRP part. Subsequently, a finite-element (FE) simulation of the cooling process was conducted to predict the spring-in phenomenon of the shaped CFRP part using the temperature data obtained in the forming simulation. Finally, a CFRP part was manufactured and compared with the results of the FE simulation.

## 1. Introduction

Recently, environmental pollution and regulations have pressured the automotive industry to improve the fuel efficiency of vehicles. Consequently, the application of several lightweight metals, such as aluminum, magnesium, and advanced high-strength steel (AHSS), has emerged as a solution for the automotive industry. Carbon fiber reinforced plastic (CFRP) has also been used in the automotive industry owing to its high performance in terms of strength and stiffness compared to lightweight metals. However, the manufacturing of CFRP parts increases production costs and induces low productivity [1,2,3]. Additionally, the prediction of dimensional and geometrical distortions is necessary to increase the demand for composite materials [4,5].

Recent studies have focused on FE simulations of several manufacturing processes to produce composite parts. Poodts et al. conducted an FE simulation of resin transfer molding (RTM) to find optimal process parameters and compared the mechanical properties of the composite parts manufactured by RTM and the autoclave processes [6]. Here, the FE simulation was composed of the injection and the curing stages to consider whole manufacturing processes. However, the RTM process is disadvantageous in cost-driven industries because of the requirement for additional tools, such as an entry tool to inject the resin. Lee et al. conducted an FE simulation of a prepreg compression molding (PCM) process similar to the stamping process and manufactured automotive CFRP parts using PCM to substitute conventional steel parts [7]. However, they focused on the design of the manufacturing process to satisfy the required strength of the composite part, except for the dimensional accuracy.

The distortion of the composite part induces an increase in the production cost and wastes time. The distortion can lead to parts not fitting correctly in an assembly process. This is particularly problematic in the aerospace, automotive, and precision engineering industries, where tight tolerances are required. Additionally, modification of the tool shape is required if the corresponding surfaces of the parts are mismatched. Therefore, distortion prediction is important in manufacturing composite parts. Bellini et al. investigated the influence of some geometrical parameters on the spring-in of a thin CFRP part [8]. FEM considering thermo-chemical and thermo-mechanical phenomena was conducted with process parameters such as the corner radius of the mold, thickness of laminate, and layup sequence. They confirmed that the only layup sequence of laminate influences the spring-in. However, their simulation was conducted considering only the curing process. Groh et al. evaluated the effect of processing parameters, such as the layup, curing temperature, cooling rate, and fiber volume fraction, to predict spring-in deformations using fast-curing epoxy resins (FCER) [9]. However, their prediction method was only applicable to L-shaped composite parts because the spring-in deformation was calculated using an analytical method. Mezeix et al. proposed a new approach to predict the distortion of parts manufactured by the autoclave process and evaluated the effect of the out-of-plane shear stress at the interface and the in-plane normal stress of the laminate [10]. However, they conducted an FE simulation of an uncoupled cooling process with forming during the autoclave process. Fiorina et al. proposed a new approach based on a global–local approach and a three-step methodology to predict the springback of composite structures manufactured by the autoclave process [11]. However, most of the manufacturing processes mentioned above are not economical because they require additional manufacturing tools, such as an entry nozzle to inject the resin and an autoclave system composed of many components. The stamping process is an alternative manufacturing process because it does not require an additional manufacturing tool. However, predicting the spring-in of the composite part manufactured through the stamping process is necessary to reduce the cost of mold modification.

The purpose of this study is to predict the spring-in deformation of composite parts manufactured by a stamping process using a coupled analysis of the forming and cooling processes. First, an FE simulation of the forming process was performed to design the stamping process of the CFRP part. Process parameters such as the initial temperature of the CFRP laminate and the mold temperature were determined to optimize the forming conditions. Second, an FE simulation of the cooling process was performed to predict the spring-in deformation of the composite part. The temperature distribution of the composite part obtained from the forming simulation was used as the initial temperature condition for the cooling process to perform the coupled analysis of the forming simulation. Finally, an L-shaped composite part was manufactured through a stamping process and compared with the results of the FE simulation for verification.

## 2. Materials and Methods

### 2.1. Mechanical Properties of CFRP at Various Temperatures

The composite material used in this study was a woven prepreg (SK Chemicals, Seongnam, Republic of Korea). The thickness of the ply was 0.25 mm, and the fiber content was evaluated as 45%. The resin was thermoplastic polyurethane with a glass transition temperature (T_g_) of 110 °C and a melting temperature (T_m_) of 146 °C.

**Figure 1:**
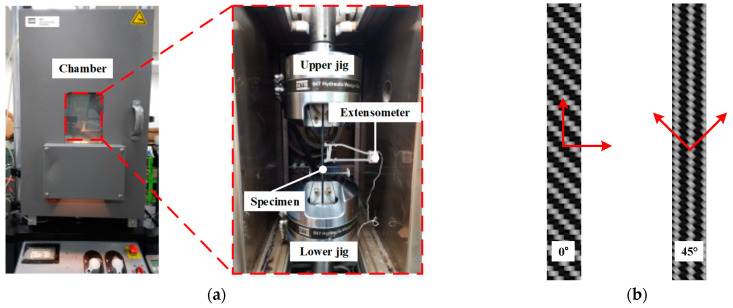
Experimental setup for tensile test of CFRP laminate at various temperatures, (**a**) Equipment for tensile test, (**b**) CFRP specimen for tensile test.

To conduct an FE simulation of forming and cooling processes during stamping, the mechanical properties of the CFRP laminate were obtained at various temperatures. A total of 40 CFRP laminate specimens were fabricated and divided into 8 groups, as shown in Table 1. Tensile tests were conducted at elevated temperatures with an MTS universal testing machine (MTS Lankmark^TM^ 100 kN, MTS Systems Corporation, Eden Prairie, MN, USA) of 10-ton capacity, as shown in Figure 1. Specimens with a thickness of 2.0 mm were tested for 0° and 45° directions according to the ASTM D3039 and ASTM D3518 standards [12,13]. The specimen was placed in a chamber for 10 min to increase the temperature. The test was conducted at 20 °C, 70 °C, 90 °C, and 110 °C to determine the characteristics of the resin at different temperatures. The test results indicated that the elastic modulus in each direction decreased as the temperature increased. In particular, the elastic modulus decreased remarkably as the material temperature approached the glass transition temperature (T_g_). The mechanical properties of the CFRP laminates at various temperatures are summarized in Table 1.

### 2.2. Thermal Properties of Tool Steel and CFRP Material

Generally, tool steel has a higher coefficient of thermal expansion (CTE) than CFRP. Therefore, the tool–part interaction owing to the difference in CTE between the tool steel and CFRP is one of the main factors inducing residual stresses in the composite part [14,15,16,17]. The thermal properties of the CFRP material, such as the thermal conductivity, specific heat, and CTE, are summarized in Table 2 according to the temperature and fiber direction. Three specimens were fabricated for tests in each experimental condition. The thermal conductivity and specific heat were measured using a laser flash analysis test, and the CTE was measured using a thermomechanical analysis test. The thermal conductivity, specific heat, and CTE of the H13 tool steel at various temperatures are summarized in Table 3. The thermal properties of both materials were employed in the FE simulation of the stamping process.

## 3. Stamping Process for the CFRP Part

### 3.1. The Procedure of Stamping Process for Manufacturing a Composite Part

**Figure 2:**
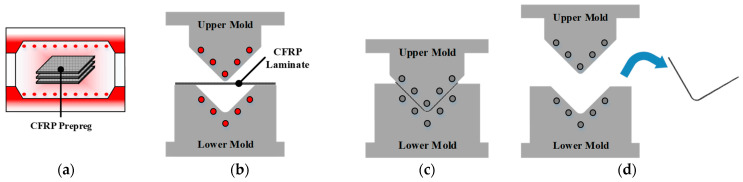
Schematic of stamping process for manufacturing composite part. (**a**) CFRP heating; (**b**) Transferring and forming; (**c**) Cooling; (**d**) Demolding.

**Figure 3:**
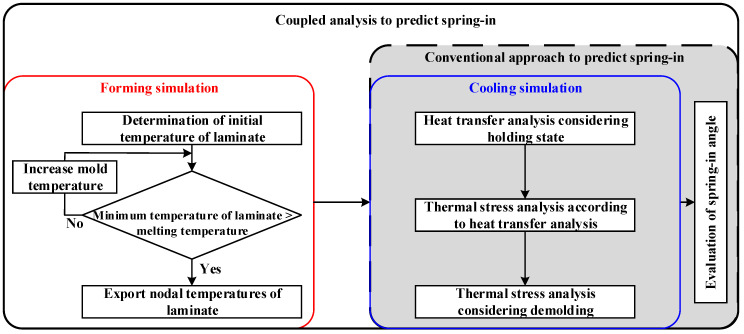
FE simulation procedure for manufacturing the composite part through the stamping process.

The RTM and autoclave processes require additional equipment, such as an entry nozzle for injecting the resin and an autoclave system, to produce the CFRP part. However, the stamping process does not require additional ones. The stamping process to produce the CFRP part is divided into four steps: (a) heating the stacked CFRP prepreg in a heating oven, (b) transforming the CFRP laminate on the lower mold and forming, (c) cooling the formed CFRP part inside the closed molds, and (d) demolding the produced part, as shown in Figure 2. Figure 3 shows the FE simulation procedure for manufacturing a composite part using the stamping process. Previous studies conducted FE simulations of cooling without considering the forming process. They conducted these simulations assuming that the mold and part were isothermal as the initial conditions. However, the initial states of the mold and part are not isothermal during the actual stamping process. In this study, a coupled analysis of the forming and cooling was conducted to consider the actual manufacturing process of stamping. First, the forming process was designed using FE software PAM-FORM 2020 to determine the temperature of the mold as a process parameter. Using the optimized results, the temperature distribution of the CFRP laminate was obtained at the end of the forming process. Second, an FE simulation of the cooling process was conducted to predict the spring-in angle of the CFRP part using FE software ABAQUS 2020. Unlike the conventional method, the simulation of the cooling process was coupled with that of the forming process, and the temperature of the CFRP laminate at the end of the forming process was employed as the initial temperature of the CFRP laminate during the cooling process. Heat transfer analysis was performed to predict the temperature variation during the cooling process of the holding state. Subsequently, a thermal stress analysis was conducted using the results of the heat transfer analysis. Finally, the thermal stress analysis of the demolding state was conducted to predict the spring-in of the composite part.

### 3.2. Process Design of Stamping Process by FE Forming Simulation

**Figure 4:**
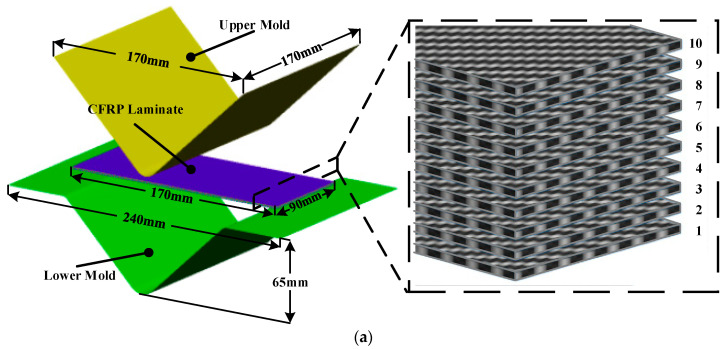

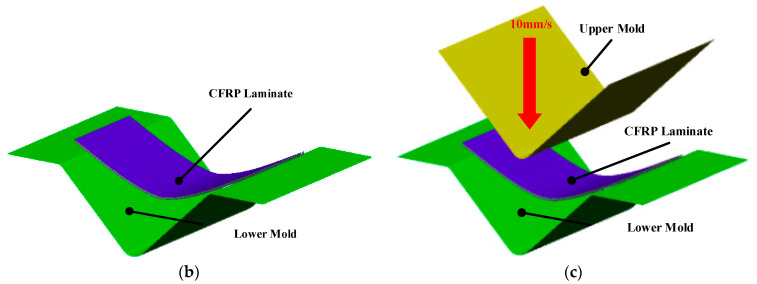
FE model of the stamping process. (**a**) Initial state; (**b**) Gravity analysis; (**c**) Forming process.

Generally, thermoplastic CFRP exhibits significant differences in material properties depending on the temperature. Therefore, the temperatures of the CFRP laminate and mold are significant process parameters in the stamping process. In this study, an FE simulation of the forming process was performed to determine the thermoforming conditions. Figure 4 shows the FE model in which the stamping process is divided into the initial state, gravity analysis, and forming process. As shown in Figure 4a, the CFRP laminate was composed of 10 prepreg plies with dimensions of 90 mm × 170 mm in [010]. The velocity of the upper mold was 10 mm/s and the lower mold was fixed, as shown in Figure 4c. In the FE model, the CFRP and molds were modeled using the 4-node rectangular shell element. Additionally, the mesh size of CFRP was 2 mm considering that the distance between fiber and fiber is 2 mm. First, the initial temperature of the CFRP laminate was determined to be 190 °C considering the transfer time. In fact, the maximum heating temperature of the CFRP material is limited to 200 °C to prevent the evaporation of thermo-polyurethane resin. However, the temperature of CFRP decreases to 190 °C when transferred from the heating oven to the mold.

**Figure 5:**
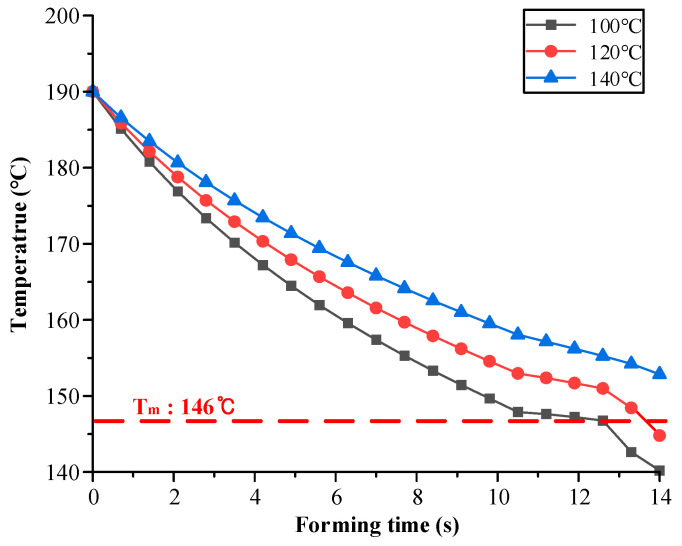
Temperature histories of the CFRP laminate during the forming process.

In general, the cooling time depends on the mold temperature during forming. A low forming temperature plays a significant role in reducing the cooling time. In this study, various temperatures such as 100 °C, 120 °C, and 140 °C were selected as the mold temperature during the forming process. The temperature history of the CFRP laminate was predicted using commercial FE software PAM-FORM 2020. The friction coefficient at the interface of the CFRP and the mold was applied to consider the interface behavior. In the case of 1 MPa pressure, the friction coefficients were determined as 0.03, 0.05, 0.09, 0.10, and 0.15 for 110 °C, 130 °C, 150 °C, 170 °C, and 190 °C, respectively. In the case of 5 MPa pressure, the friction coefficients were determined as 0.01, 0.02, 0.05, 0.06, and 0.07 for 110 °C, 130 °C, 150 °C, 170 °C, and 190 °C, respectively [7]. In addition, the interfacial heat transfer coefficients of the CFRP and mold were employed to predict the heat loss of the composite laminate. The heat transfer coefficients were determined as 59 W/m^2^·°C, 65 W/m^2^·°C, and 72 W/m^2^·°C for 2.0 MPa, 3.5 MPa, and 5.0 MPa, respectively [19]. Figure 5 shows the minimum temperature history of the CFRP laminates during the forming process. In the case of mold temperatures of 100 °C and 120 °C, the results showed that the minimum temperature of the CFRP laminate decreased to below Tm owing to the heat transfer between the mold and CFRP laminate. However, when the temperature of the mold was higher than 140 °C, the minimum temperature of the CFRP laminate was maintained above T_m_ until the forming process was completed. Tatsuno et al. suggested that CFRP sheets should be maintained at a formable temperature such that the resin becomes molten [20]. From the simulation, the optimal mold temperature was determined to be 140 °C for the forming process.

### 3.3. FE simulation of a Cooling Process to Predict Spring-In of CFRP Part

The actual manufacturing of CFRP parts is a continuous process composed of forming and cooling processes. Therefore, an FE simulation of the cooling process must be coupled with a forming simulation to consider the entire manufacturing process.

**Figure 6:**
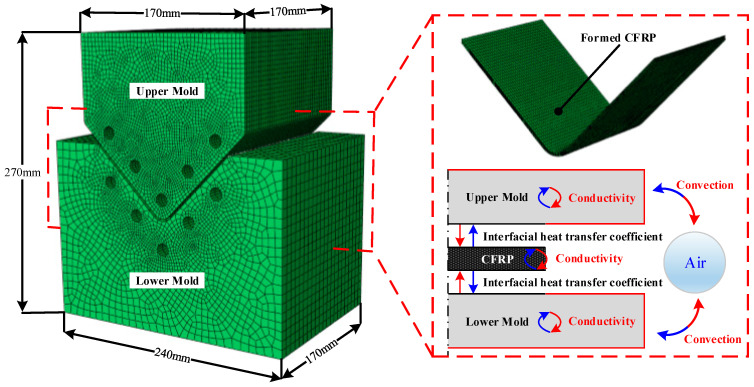
FE model and boundary conditions of the cooling process considering holding state.

**Figure 7:**
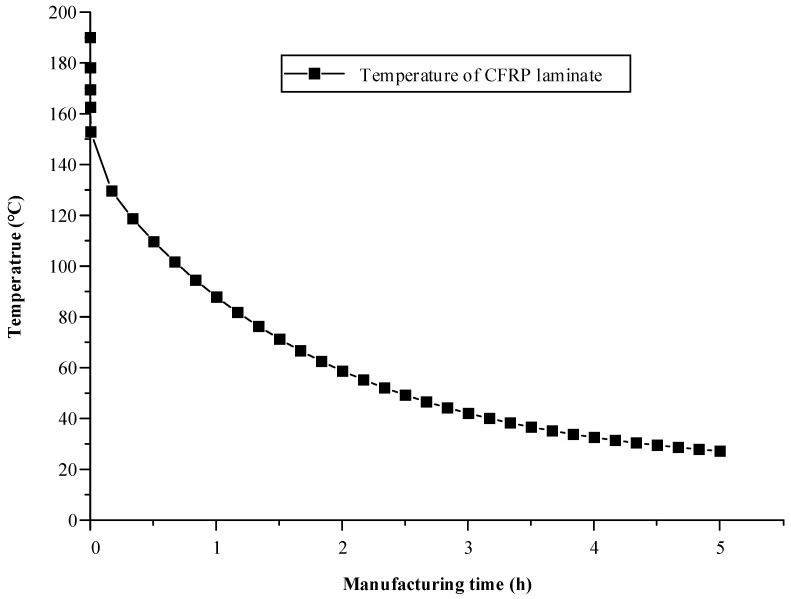
Result of heat transfer analysis considering the entire manufacturing process.

In this study, FE simulations were performed for the holding and demolding states during the cooling process. First, a heat transfer analysis of the cooling process, considering the holding state, was performed using the commercial software ABAQUS 2020. Figure 6 shows the FE model and boundary conditions of the heat transfer analysis performed considering the cooling process. The FE model was composed of an upper mold, a lower mold, and the formed CFRP part. The FE model of the CFRP part was exported from the forming simulation software. Additionally, each mold was modeled as an elastic body in the FE simulation to consider thermal shrinkage during the cooling process. In the FE model, the molds were modeled using the 8-node linear brick. Additionally, the mesh size of the molds was adjusted from 0.5 mm to 10 mm to improve the convergence of the simulation. The initial temperature of both molds was 140 °C, and the nodal temperatures of the CFRP laminate at the end of the forming simulation were employed as the initial temperature distribution of the CFRP part for the heat transfer analysis. The FE model used various thermal properties of the CFRP and tool materials, as listed in Table 2 and Table 3. The upper and lower molds were kept closed until the cooling process, considering the holding state, was complete. Therefore, the temperature variation in the CFRP part depends on the mold temperature. Both molds were cooled via convection, and the CFRP part was cooled via interfacial heat transfer with both molds during the cooling process. Figure 7 shows the minimum temperature histories of the CFRP part predicted by the heat transfer analysis, which is a sequentially coupled analysis of the forming and cooling processes.

**Figure 8:**
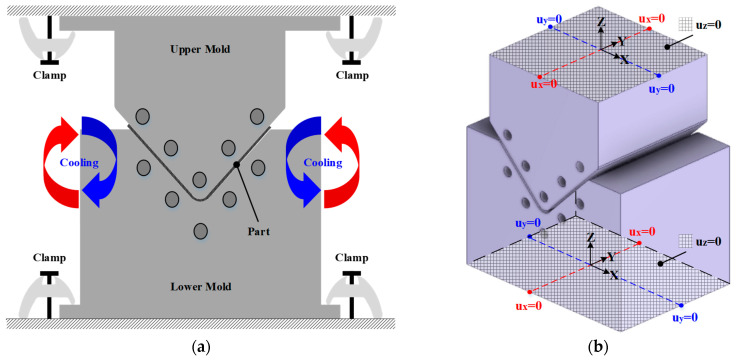
Boundary conditions and results of thermal stress analysis. (**a**) Schematic of cooling; (**b**) Boundary conditions of holding state.

A thermal stress analysis of the cooling process was performed using the result of the heat transfer analysis. The upper and lower molds were clamped to the press equipment during cooling, as shown in Figure 8a. Therefore, the top surface of the upper mold and the bottom surface of the lower mold were imposed to the fixed boundary conditions, as shown in Figure 8b. As mentioned in Section 2.2, residual stress occurs because of the tool–part interaction during the cooling process. Therefore, the friction coefficient was applied to consider the residual stress due to the shearing of the part and molds. When the CFRP part was ejected from the mold, shape deformation occurred because of generated residual stress during the cooling process. Therefore, a thermal stress analysis considering the demolding state was conducted to predict the final shape of the CFRP part. The upper and lower molds were not considered to be demolding because the part was ejected.

### 3.4. Comparison of Coupled Analysis and Conventional Approach Results

**Figure 9:**
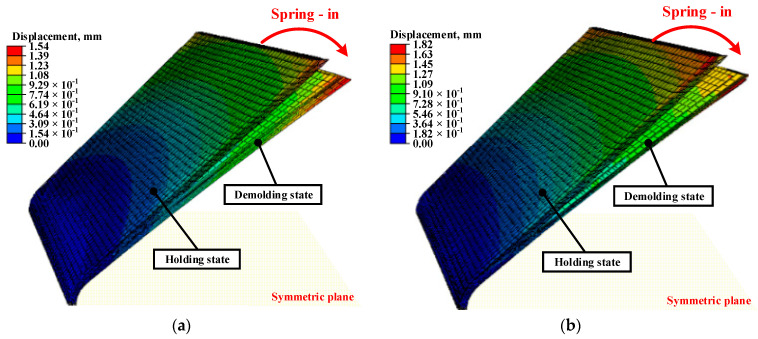
Total deformation values predicted by FE simulation. (**a**) Proposed method; (**b**) Conventional approach.

A coupled analysis of the forming and cooling processes was performed to consider the entire manufacturing process of the composite parts. However, other studies assumed isothermal conditions for the molds and parts. In other words, they did not conduct forming simulations for several reasons. To verify the necessity of the coupled analysis, an FE simulation was conducted using a conventional approach. All procedures of the FE simulation were the same as those mentioned above, except that the forming simulation was performed. The cooling process of the coupled analysis employed the result of forming as initial conditions, such as the mold temperature and temperature distribution of the CFRP part. Hence, the temperature gradient in in-plane and thickness direction was applied to the CFRP part. However, in the case of a conventional approach, the isothermal condition was applied at 150 °C to the CFRP part, upper mold, and lower mold as the initial conditions considering that the forming should be completed at a higher temperature than T_m_. The FE results can be different due to the temperature difference of molds and the application of the temperature gradient on the CFRP part. Subsequently, heat transfer and thermal stress analyses of the cooling process were conducted to obtain the final shape of the CFRP. The deformation of the coupled analysis was smaller than the conventional one, as shown in Figure 9. In the case of the coupled analysis, the mold temperature was lower than the conventional one. This means that the mold shrinkage of the conventional approach was increased as compared with that of the coupled analysis. The difference in mold temperature between two methods in FE analysis would contribute to the occurring residual stress of the CFRP part.

## 4. Experimental Verification

### 4.1. Manufacturing of the CFRP Part through the Stamping Process

**Figure 10:**
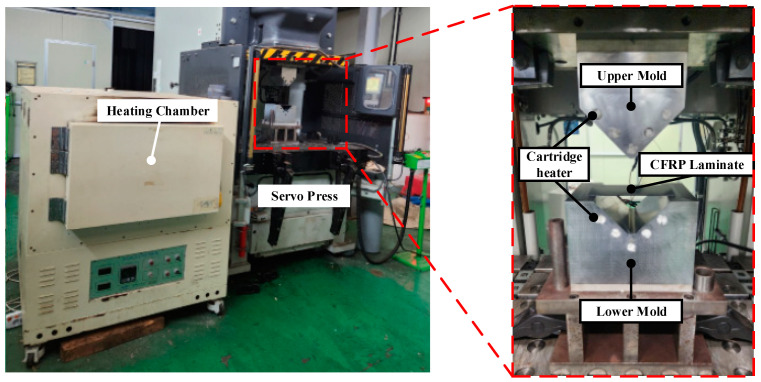
Experimental equipment for manufacturing the CFRP part.

In this study, experiments were conducted to validate the proposed method under the same conditions as those used in the FE simulation. Figure 10 shows the equipment used to manufacture the CFRP parts using the stamping process. The upper and lower molds with an inserted cartridge heater were placed on a 2000-kN servo press. A heating chamber was used to heat the CFRP laminate to 200 °C. The thickness of the CFRP laminate with 10 plies was 2.5 mm, as described in Section 3.2. The CFRP part was manufactured using a mold temperature of 140 °C, similar to the condition in the FE simulation.

The manufacturing process of the CFRP part was as follows: First, the liquid release agent was spread on the surfaces of the upper and lower molds to prevent the CFRP laminate from sticking to each mold. Subsequently, the CFRP laminate was heated to 200 °C in the heating chamber. The heated CFRP laminate was then transferred to a heated lower mold. The upper mold was descent to conduct stamping after the CFRP laminate was positioned on the lower mold. Finally, the CFRP part was ejected after cooling for 5 h.

### 4.2. Comparison of Experiment and FE Simulation Results

**Figure 11:**
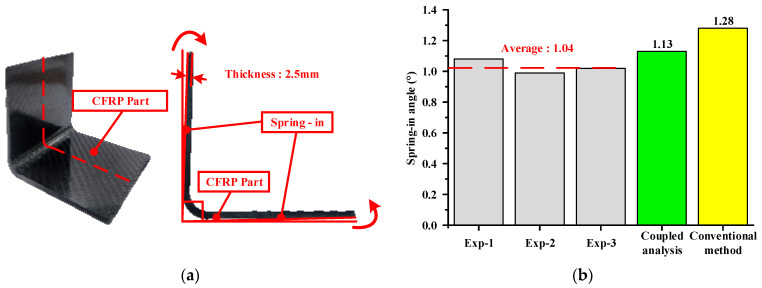
Experimental results of manufacturing through the stamping process. (**a**) Experiment result; (**b**) Comparison of experiment and FEM.

In this study, the experimental and FE results for the spring-in angle were compared to verify the proposed approach. The shape of the experimental results was measured using 3D scanning equipment, and the results are shown in Figure 11a. Figure 11b presents a comparison between the experimental and FE results. The average spring-in angle in the experiment results was measured at 1.04°. Moreover, the spring-in angles of the coupled analysis and conventional methods were 1.13°and 1.28°, respectively. The prediction error of the proposed method was 8.65%, whereas that of the conventional method was 23.07%. Based on the comparison, the coupled analysis of the forming and cooling processes was more suitable than the conventional approach neglecting the forming simulation. This indicates that the forming simulation has a considerable influence on the spring-in angle of the CFRP part. Therefore, the proposed FE simulation approach is a more reasonable method for predicting the spring-in angle of CFRP parts manufactured through the stamping process.

The proposed approach is helpful in designing the manufacturing process and mold shape. In general, the composite material is deformed at a high temperature and consolidated during the cooling process to produce the final product. FE simulation is useful in optimizing the process parameters, such as mold velocity, mold temperature, and initial temperature of the CFRP blank, and for reducing the manufacturing time. In this study, the mold temperature was lower than those in the conventional method. Nevertheless, the occurrence of residual stress due to temperature change is inevitable, which will induce distortion. Therefore, the modification of the mold shape is essential to satisfy the dimensional accuracy. In this study, the result of the proposed approach was more accurate than the conventional approach. In addition, the coupled analysis was conducted with only the given geometry CAD files and manufacturing conditions. Thus, it can potentially be applied to other shapes. The proposed method can be used to determine the optimal mold shape and help to reduce the trial and error.

## 5. Conclusions

In this study, the spring-in angle of a CFRP part manufactured by stamping was predicted by FE simulation and validated by the experimental implementation. A procedure for the coupled analysis of the forming and cooling processes to predict the spring-in angle was proposed, and the results were as follows: First, various mold-forming temperatures were evaluated to determine the optimal temperature via FE simulation. This is because the thermoplastic CFRP material must be maintained above the Tm of the resin until the forming process is complete. At a mold temperature of 140 °C, the minimum temperature of the CFRP laminate was maintained above Tm during forming. An FE simulation of the cooling process was conducted to predict the spring-in of the CFRP part. The nodal temperature distribution of the CFRP laminate at the end of forming was employed as the initial condition for the heat transfer analysis to conduct a coupled analysis of the forming and cooling processes. A heat transfer analysis was performed to obtain the temperature variations in the CFRP parts and molds. A thermal stress analysis of the holding state was conducted using the results of the heat transfer analysis. Generally, spring-in occurs because of the generation of residual stress during cooling. In the final step of the simulation, a thermal stress analysis considering the demolding state was conducted to predict the deformed shape of the CFRP part. Additionally, a simulation of the cooling process using the conventional method was performed for comparison with the proposed method. The FE results of the coupled analysis and conventional method predicted different values. To verify the necessity of a coupled analysis of the forming and cooling processes, a CFRP part was manufactured using the stamping process. The prediction error of the proposed method was 8.65%, whereas that of the conventional method was 23.07%. A comparison of the experimental and FE results indicated that the FE results obtained using coupled analysis were more accurate. Therefore, the method proposed in this study is appropriate for predicting the spring-in behavior of CFRP parts manufactured via stamping. 

## Figures and Tables

**Table 1:** Mechanical properties of CFRP laminate at various temperatures.

Mechanical Properties	Symbol	Temperatures	No. of Specimen	Values
Elastic modulus in fiber direction, transverse direction	E_11_ = E_22_	20 °C	5	72.30 GPa
		70 °C	5	55.04 GPa
		90 °C	5	16.98 GPa
		110 °C	5	4.01 GPa
Shear modulus in 1–2 plane	G_12_	RT	5	3.99 GPa
		70 °C	5	2.32 GPa
		90 °C	5	0.19 GPa
		110 °C	5	0.04 GPa

**Table 2:** Thermal properties of CFRP material.

Density (g/cm^3^)	Thermal Conductivity (W/m °C)		Specific Heat (J/kg °C)		Coefficient of Thermal Expansion (10^−6^/°C)		Temperature (°C)
	Longitudinal	Thickness	Longitudinal	Thickness	Longitudinal	Thickness	
1.52	3.906	0.630	1283	1516	7.75	29.45	80
	4.043	0.632	1515	1757			120

**Table 3:** Thermal properties of H13 tool steel [18].

Density (g/cm^3^)	Thermal Conductivity (W/m °C)	Specific Heat (J/kg °C)	Coefficient of Thermal Expansion (10^−6^/°C)	Temperature (°C)
7.65	29.5	447	10.3	25
7.65	30.3	453	11.2	100
7.58	37	502	12.1	400

## Data Availability

Data are contained within the article.
